# Detection of *Legionella*, *L. pneumophila* and Mycobacterium Avium Complex (MAC) along Potable Water Distribution Pipelines

**DOI:** 10.3390/ijerph110707393

**Published:** 2014-07-18

**Authors:** Harriet Whiley, Alexandra Keegan, Howard Fallowfield, Richard Bentham

**Affiliations:** 1 Health and the Environment, Flinders University, GPO Box 2100, Adelaide 5001, Australia; E-Mails: Howard.Fallowfield@flinders.edu.au (H.F.); Richard.Bentham@flinders.edu.au (R.B.); 2 South Australian Water Corporation, 250 Victoria square, Adelaide 5000, Australia; E-Mail: Alex.Keegan@sawater.com.au

**Keywords:** *Legionella*, *L. pneumophila*, Mycobacterium avium complex (MAC), Nontuberculous Mycobacteria (NTM), potable water, distribution systems, public health

## Abstract

Inhalation of potable water presents a potential route of exposure to opportunistic pathogens and hence warrants significant public health concern. This study used qPCR to detect opportunistic pathogens *Legionella* spp., *L. pneumophila* and MAC at multiple points along two potable water distribution pipelines. One used chlorine disinfection and the other chloramine disinfection. Samples were collected four times over the year to provide seasonal variation and the chlorine or chloramine residual was measured during collection. *Legionella* spp., *L. pneumophila* and MAC were detected in both distribution systems throughout the year and were all detected at a maximum concentration of 10^3^ copies/mL in the chlorine disinfected system and 10^6^, 10^3^ and 10^4^ copies/mL respectively in the chloramine disinfected system. The concentrations of these opportunistic pathogens were primarily controlled throughout the distribution network through the maintenance of disinfection residuals. At a dead-end and when the disinfection residual was not maintained significant (*p* < 0.05) increases in concentration were observed when compared to the concentration measured closest to the processing plant in the same pipeline and sampling period. Total coliforms were not present in any water sample collected. This study demonstrates the ability of *Legionella* spp., *L. pneumophila* and MAC to survive the potable water disinfection process and highlights the need for greater measures to control these organisms along the distribution pipeline and at point of use.

## 1. Introduction

The presence of pathogenic organisms in potable water constitutes a significant public health risk [1]. This includes not only enteric pathogens such as noroviruses, *Cryptosporidium* spp. and *Campylobacter* spp.; but also opportunistic pathogens such as *Legionella* spp., Non-tuberculous Mycobacterium (NTM), *Pseudomonas aeruginosa* and *Acanthamoeba* spp. [1,2,3]. Current public health guidelines primarily focus on the control of enteric pathogens and indicator organisms to monitor microbial water quality [4]. Consistently, studies have demonstrated a lack of correlation between opportunistic pathogens and indicator organisms [5,6,7]. This is of concern as opportunistic pathogens are fast becoming the primary cause of waterborne disease in developed countries [2].

*Legionella* spp. are the causative agent of Legionelloses, including Legionnaire’s disease a serious atypical pneumonia infection, and Pontiac fever, an acute febrile illness [8]. Legionellosis is primarily caused by inhalation of contaminated aerosols and was first associated with potable water in 1980 when *Legionella* isolates from patients in a renal graft unit were identified as similar to strains isolated from shower-bath mixers in the same unit [9]. Recently a significant increase in the incidence of Legionellosis has been observed in the United States [10] and across Europe (GIDEON Global infectious disease and epidemiology network, 2012). In the USA from 2009–2010 *Legionella* spp. were responsible for 58% of USA drinking water related disease outbreaks reported to the CDC [11].

NTM refers to Mycobacteria distinct from the *Mycobacterium tuberculosis* complex [12]. NTM have been identified in drinking water systems, hospital distribution systems and domestic tap water [13]. In recent years an increase in the incidence of NTM pulmonary disease has been reported in many parts of the world [14]. One of the most common NTM associated with human disease is Mycobacterium avium complex (MAC) which includes *M. avium* (*M. avium* subspecies *avium* (MAA), *M. avium* subspecies *hominis* (MAH), *M. avium* subspecies *paratuberculosis* (MAP)) and *Mycobacterium* *intracellulare* [15]. In Australia 74% of all non-AIDS related NTM cases are due to MAC [16]. The routes of MAC infection are via inhalation or ingestion of MAC contaminated materials. The complex is responsible for a wide range of illnesses including fibrocavitary lung disease [17], fibronodular bronchiectasis [18], pulmonary nodules simulating lung cancer [19], hypersensitivity pneumonitis [20], cutaneous skin [21] and soft tissue infection [22], cervical lymphadenitis in children [23], gastrointestinal tract and disseminated infection in immune compromised patients [24], and putatively Crohn’s disease [25], cited in Whiley *et al.* [26].

Numerous studies have linked MAC infections to potable water sources including hospital water distribution systems [27,28,29] and domestic hot water systems [30]. Notwithstanding, there is a paucity of studies addressing the presence of MAC in Australian potable water and routine monitoring/testing for *Mycobacteria* and *Legionella* in potable water is not mandated by Australian public health guidelines [13,31]. In this paper, qPCR was used to investigate and compare the presence of *Legionella* spp., *L. pneumophila* and MAC along two South Australian potable water distribution pipelines. One system utilizing chlorine disinfection and the other chloramine disinfection. The influence of temperature, chlorine or chloramine disinfection residual and distance along the pipeline from the water treatment plant on the concentrations of MAC and *Legionella* were assessed. The results of this investigation may permit a preliminary assessment of the possible exposure of the population to these organisms via the potable water supply.

## 2. Experimental Section

**Figure 1:**
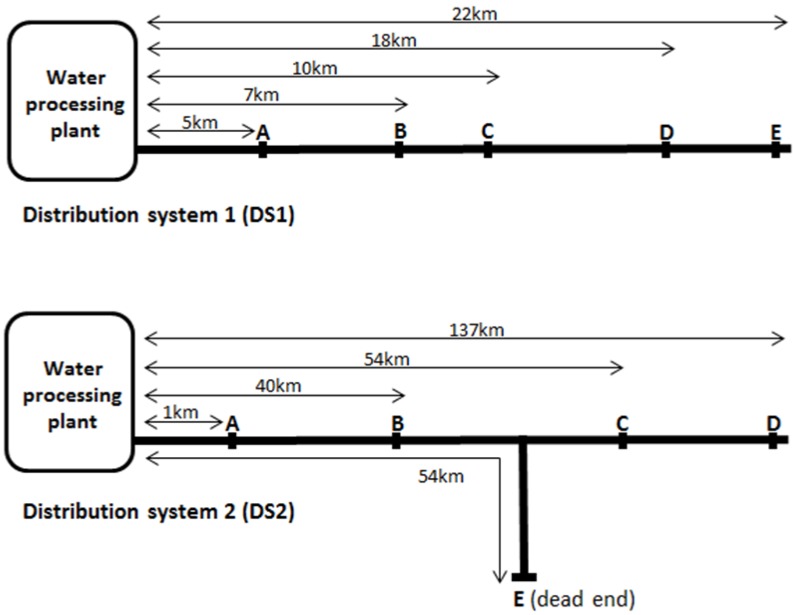
Schematic showing the sampling points in distribution system 1 (DS1) and distributions system 2 (DS2). Schematic is not too scale.

Samples were collected aseptically, using the AS/NZS 5667 standard method for water quality sampling, from multiple points along two South Australian potable water distribution networks, shown in Figure 1. It is important to note distribution system 2 is significantly longer than distribution system 1. Sampling was repeated four times over the year, once during summer (February), autumn (May), winter (August) and spring (November) (South Australia has a Mediterranean climate with warm summers and cool winters). The water temperature during each sampling periods was measured and provided by the water utility company. Water within Distribution System 1 (DS1) was treated with coagulation, flocculation, sedimentation, full-flow micro-filtration and disinfection with chlorine whereas water within Distribution System 2 (DS2) was treated with coagulation, flocculation, sedimentation, sand filtration, disinfection with ultra violet light and chloramine. At each sampling point the total and free chlorine or monochloramine residual was measured and 500 mL water samples collected in triplicate. The chlorine or monochloramine present in the samples was quenched with excess sodium thiosulphate and samples stored at 4 °C for up to 12 h before biological analysis and DNA extraction. *Escherichia coli* and total coliforms were enumerated with Colilert^TM^ trays (IDEXX Laboratories) using the standard method.

DNA was extracted for qPCR analysis from 450 mL of the sampled water using the BIO-RAD Aquadien™ Kit following manufacturer’s instructions giving a final volume of 100 µL of DNA extract (Bio-Rad Laboratories, Inc., Sydney, NSW, Australia). Triplicate qPCR was then performed for the enumerations of *Legionella* spp., *L. pneumophila* and MAC.

*Legionella* spp. qPCR was performed as previously described [32]. The 25 µL reaction volume contained 1× PCR buffer (Invitrogen), 2.5 mM MgCl_2_ (Invitrogen), 2.5 mM SYTO9 fluorescent dye (Invitrogen), 0.2 mM deoxynucleoside triphosphate mix (Invitrogen), I U platinum Taq DNA polymerase (Invitrogen), 0.3 µM JFP primer (5'-AGGGTTGATAGGTTAAGAGC-3'), 0.3 µM JRP primer (5'-CCAACAGCTAGTTGACATCG-3') and 5 µL template DNA. The cycling conditions included an initial hold at 95 °C for 5 min, followed by 45 cycles consisting of 94 °C for 10 s, 60 °C for 20 s, and 72 °C for 20 s.

*L. pneumophila* qPCR was also performed as previously described [33]. The reaction volume was 25 µL and included 1× PCR buffer (Invitrogen), 2.5 mM MgCl_2_ (Invitrogen), 2.5 mM SYTO9 fluorescent dye (Invitrogen), 0.2 mM deoxynucleoside triphosphate mix (Invitrogen), I U platinum Taq DNA polymerase (Invitrogen), 0.5 µM mip99F primer (5'-TGTCTTATAGCATTGGTGCC-3'), 0.5 µM mip213R primer (5'-CAATTGAGCGCCACTCATAG-3') and 5 µL of template DNA. The cycling conditions included an initial hold at 95 °C for 5 min, followed by 40 cycles consisting of 94 °C for 20 s, 60 °C for 20 s, and 72 °C for 25 s.

MAC qPCR was performed using previously described primers MACF primer (5'-CCCTGAGACAACACTCGGTC-3') and MACR primer (5'-ATTACACATTTCGATGAACGC-3') [34]. The 25 µL reaction volume contained 1× PCR buffer (Invitrogen), 2.5 mM MgCl_2_ (Invitrogen), 2.5 mM SYTO9 fluorescent dye (Invitrogen), 0.2 mM deoxynucleoside triphosphate mix (Invitrogen), IU platinum Taq DNA polymerase (Invitrogen), 0.3 µM MACF primer, 0.3 µM MACR primer and 5 µL of template DNA. The cycling conditions included an initial hold at 95 °C for 5 min, followed by 45 cycles consisting of 94 °C for 15 s, 50 °C for 30 s, and 72 °C for 20 s.

All qPCR reactions were carried out in a RotorGene 3000 (Corbett Research, Sydney, Australia) with data acquisition at 72 °C on the 6-carboxyfluorescein channel (excitation at 470 nm, detection at 510 nm) at a gain of 5. Melt curve data was also acquired on this channel at gains of 2 and 5 using a ramping rate of 1 °C/60 s from 75 °C to 95 °C. Each qPCR run included a positive control and a non-template control of nuclease free water. For each reaction the melt curve was analysed and a positive *Legionella* spp., *L. pneumophila* and MAC was confirmed with a melting temperature (T*_m_*) of 88 ± 1 °C, 82.5 ± 1 °C, 85 ± 1 °C respectively.

To determine the presence of environmental inhibitors in the extracted DNA, the qPCR reactions were conducted in triplicate for both undiluted DNA extract and 1:10 dilution UltraPure^TM^ DNase/RNase-Free Distilled Water (Invitrogen) of the same sample. If the cycle threshold (C*_T_*) value for the 1:10 dilution of DNA extract was less than approximately 3.3 (representing approximately 1-log_10_ concentration value) [35] than the pure DNA extract then it was assumed that environmental inhibitors were present. When inhibition was present in the undiluted DNA extract and the 1:10 dilution had the correct T*m* this was used to calculate copies/mL.

Standard curves were created using positive PCR product purified using a Montage PCR Centrigual Filter Device (Millipore, Melbourne, VIC, Australia) following the manufacturer’s instructions. The concentration of purified DNA was calculated by reading the absorbance spectrophotometrically at 260 nm and 280 nm. The number of copies of PCR product was determined using the URI Genomics and Sequencing Center, calculator for determining the number of copies of a template available at http://cels.uri.edu/gsc/cndna.html [36]. Then a 1:10 series of dilutions (ranging from 10^9^–10^0^ copies) were created using the Corbett Research liquid handling system (Corbett Research, Sydney, Australia). This was used to determine both the limit of detection of each assay and the calculated copies. If amplification was not detected or the melt curve was incorrect the sample was allocated a value of half the limit of detection. If a sample contained multiple melt peaks, that included the correct one this value was not included.

Statistical analysis of average calculated copies/mL of each organism at the different sampling points, time periods and distribution systems was conducted using Graph Pad^TM^ prism 5.0 (Graph Pad software Inc., Ambion Foster City, CA, USA). Comparisons of the average calculated copies were performed using a one-way ANOVA with Bonferroni *post hoc* test, statistical significance was accepted at *p* < 0.05.

## 3. Results

*E. coli* and total coliforms were not detected at any time throughout either distribution system. Using qPCR, *Legionella*, *L. pneumophila* and MAC were detected in both distribution systems throughout the year. Within each season, the concentration of *Legionella*, *L. pneumophila* and MAC measured at the sampling points A, closest to each respective water treatment plant as shown in Figure 1, were not statistically significantly different (*p* < 0.05) between the two distribution systems. Within the pipelines, *Legionella*, *L. pneumophila* and MAC were all detected at a maximum concentration of 10^3^ copies/mL for the chlorine disinfected DS1 and 10^6^, 10^3^ and 10^4^ copies/mL respectively for the chloramine disinfected DS2.

The average concentrations of each organism, the season that the sample was collected, the distance from the processing plant, average water temperature and chlorine or monochloramine residual are shown in Table 1 for DS1 and Table 2 for DS2. The samples highlighted indicate where a significantly (*p* < 0.05) higher concentration of an organism was detected compared to the concentration of the organism measured at sample point A for the same sampling time period. In DS1 and DS2 throughout the year there are a total of 16 water samples collected (not including sample point A) for each of these, 3 organisms were enumerated. A statistically significant increase in an organism’s concentration when compared with sample point A was observed twice in DS1 and four times for DS2 and a non statistically significant increase in magnitude was observed three times in DS1 and once in DS2.

**Table 1:** Average concentration of *Legionella* spp. *L. pneumophila* and MAC (mean ± standard deviation copies/mL) measured at each sampling point of Distribution system 1 (DS1) using qPCR. Total and free chlorine (mg/L) measured when samples were collected is also shown as well as the average water temperature for the month during which the sample was taken. The sampling points where a significant increase (*p* < 0.05) in the concentration of an organism compared to the concentration measured at sample point A within the same sampling period are also highlighted (*).

Season Sampled and Average Water Temperature	Sample Point	A	B	C	D	E
	Distance from treatment plant (km)	5	7	10	18	22
Summer 4.3 °C (*n* = 8)	Total Chlorine (mg/L)	1.4	0.7	1.1	0.4	0.4
	Free chlorine (mg/L)	1.2	0.6	0.1	0.2	0.2
	Average *Legionella* spp. (copies/mL)	37 ± 53	9 ± 4	3 ± 0	187 ± 22	^+^ 1238 ± 47
	Average *L. pneumophila* (copies/mL)	10 ± 8	3 ± 0	3 ± 0	375 ± 305	* 1981 ±298
	Average MAC (copies/mL)	36 ± 19	42 ± 19	* 31,813 ± 17,017	116 ± 118	^+^ 4395 ± 2176
Autumn 18.6 °C (*n* = 8)	Total chlorine (mg/L)	1.5	N/A	1.0	0.8	1.1
	Free chlorine (mg/L)	1.3	N/A	0.8	0.6	0.9
	Average *Legionella* spp. (copies/mL)	5 ± 4	N/A	41 ± 21	47 ± 13	46 ± 17
	Average *L. pneumophila* (copies/mL)	3 ± 0	N/A	3 ± 0	46 ± 68	^+^ 487 ± 406
	Average MAC (copies/mL)	25 ± 0	N/A	25 ± 0	200 ± 157	25 ± 0
Winter 13.6 °C (*n* = 8)	Total chlorine (mg/L)	1.2	N/A	1.3	0.4	0.7
	Free chlorine (mg/L)	1.1	N/A	1.3	0.3	0.6
	Average *Legionella* spp. (copies/mL)	22 ± 31	N/A	3 ± 0	5 ± 1	3 ± 0
	Average *L. pneumophila* (copies/mL)	81 ± 134	N/A	3 ±1	3 ± 0	3 ± 0
	Average MAC (copies/mL)	25 ±0	N/A	25 ± 0	25 ± 0	25 ± 0
Spring 20.3 °C (*n* = 8)	Total chlorine (mg/L)	1.0	0.7	0.3	0.4	0.7
	Free chlorine (mg/L)	0.9	0.6	0.2	0.3	0.6
	Average *Legionella* spp. (copies/mL)	43 ± 69	120 ± 151	8 ± 3	93 ± 139	6 ± 6
	Average *L. pneumophila* (copies/mL)	36 ± 34	15 ± 12	9 ± 1	166 ± 122	3 ± 0
	Average MAC (copies/mL)	2468 ± 317	2224 ± 2342	1112 ± 328	294 ± 58	101 ± 69

N/A sample was not available to be collected at this time. * statistically significant increase. ^+^ an increase of concentration by an order of magnitude. The lack of statistical significance (*p* > 0.05) is possible due to the large variance in environmental samples shown by the standard deviation.

**Table 2:** Average concentration of *Legionella* spp. *L. pneumophila* and MAC (mean ± standard deviation copies/mL) measured at each sampling point of Distribution system 2 (DS2) using qPCR. Total monochloramine (mg/L) measured when samples were collected is also shown as well as the average water temperature for the month during which the sample was taken. The sampling points where a significant increase (*p* < 0.05) in the concentration of an organism compared to the concentration measured at sample point A within the same sampling period are also highlighted (*).

Season Sampled and Average Water Temperature	Sample Point	A	B	C	D	E
	Distance from treatment plant (km)	1	40	54	137	54
Summer 27.3 °C (*n* = 10)	Monochloramine (mg/L)	3.6	3.0	3.6	2.7	<0.05
	Average *Legionella* spp. (copies/mL)	444 ± 96	161 ± 19	134 ± 94	423 ± 399	* 316,956 ± 169,8982
	Average *L. pneumophila* (copies/mL)	105 ± 8	712 ± 158	479 ± 177	12 ± 16	941 ± 154
	Average MAC (copies/mL)	9755 ± 7808	6910 ± 6128	2803 ± 584	1739 ± 539	5362 ± 1612
Autumn 14.7 °C (*n* = 13)	Monochloramine (mg/L)	3.8	2.3	2.4	1.5	<0.05
	Average *Legionella* spp. (copies/mL)	^#^ 24,238 ± 2918	260 ± 10	24 ± 19	1597 ± 600	1094 ± 284
	Average *L. pneumophila* (copies/mL)	238 ± 232	87 ± 14	26 ± 40	666 ± 73	333 ± 86
	Average MAC (copies/mL)	542 ± 103	663 ± 325	4068 ± 1193	586 ± 0	^+^ 1424 ± 482
Winter 13.0 °C (*n* = 9)	Monochloramine (mg/L)	3.6	3.7	2.3	0.8	0.2
	Average *Legionella* spp. (copies/mL)	2016 ± 60	883 ± 143	303 ± 34	197 ± 99	* 17,7727 ± 10,2437
	Average *L. pneumophila* (copies/mL)	248 ± 31	566 ± 220	573 ± 133	281 ± 185	* 3176 ± 1950
	Average MAC (copies/mL)	367 ± 395	277 ± 144	4228 ± 3607	433 ± 271	^+^ 9526 ± 3271
Spring 21.2 °C (*n* = 10)	Monochloramine (mg/L)	2.0	2.8	3.9	2.1	<0.05
	Average *Legionella* spp. (copies/mL)	913 ± 88	1780 ± 251	914 ± 48	1111 ± 1359	* 128,9587 ± 53,042
	Average *L. pneumophila* (copies/mL)	10 ± 2	3 ± 0	4 ± 2	38 ± 31	19 ± 17
	Average MAC (copies/mL)	5184 ± 1464	2577 ± 483	2507 ± 1615	2039 ± 475	11,445 ± 3478

N/A sample was not available to be collected at this time. * statistically significant increase. ^+^ an increase of concentration by an order of magnitude. The lack of statistical significance (*p* > 0.05) is possible due to the large variance in environmental samples shown by the standard deviation. ^#^ magnitude higher, assumed to be due to biofilm fragment.

Table 1 shows that in the chlorine disinfected DS1 a statistically significant (*p* < 0.05) increase in *L. pneumophila* and MAC and a decrease in chlorine residual was observed during the summer time point of sampling. Although the concentration of *Legionella* spp. detected at sampling point E was not significantly different (*p* > 0.05) to that measured a point A there was still a magnitude increase. The lack of statistical significance was possibly due to the small sample size and the variability (shown by the standard deviation) due to environmental samples and should not detract from the public health significance of a magnitude of increase in *Legionella* concentration given the logarithmic action approach to these organisms adopted by most guidelines [37]. Bearing this in mind the significance of the *p* value should not negate the significance of an effect in terms of human health and regulation [38]. Although a similar decrease in the chlorine residual was observed during the winter and spring sampling periods there were no increases in *Legionella*, *L. pneumophila* or MAC concentrations. This suggests that the combination of low chlorine residual and warmer water at the summer time point may have resulted in the increased detections of *L. pneumophila* and MAC observed.

In Distribution system 2, sample point E, is the location of a dead-end where the monochloramine residual was not maintained. Table 2 shows that *Legionella* spp. and *L. pneumophila* were shown to significantly increase (*p* < 0.05) only at this sampling point. An increase in magnitude of MAC concentration was also observed at sample point E during the sampling that occurred in autumn and winter. A significant increase in organism concentrations did not occur at sample point C which is also 54 km from processing plant but not a dead-end. This suggests that the environmental conditions occurring at the dead-end are promoting growth. The significant increases in *Legionella*, *L. pneumophila* or MAC concentrations at sample point E occurred during summer, winter and spring suggesting that water temperature was not the dictating factor for these increases. Sample point E is 54 km away from the processing plant; however, no increase in *Legionella* spp. was observed at sample point D which is 137 km from the processing plant. The monochloramine residual was maintained along the pipeline prior to sample point D; however, it was not maintained prior to sample point E.

## 4. Discussion

This study detected *Legionella*, *L. pneumophila* and MAC in two South Australian potable water distribution systems, but failed to detected *E. coli* or total coliforms. The absence of these indicator organisms further supports previous studies which suggest that monitoring of these organisms alone is not sufficient for determining the presence or absence of potential public health risks [4].

This study used qPCR to detect *Legionella*, *L. pneumophila* and MAC due to the difficulties with culturing *Legionella* spp., specifically with the possible presence of viable but non-culturable cells (VBNC) [39]. *Legionella* spp. have been shown to become VBNC in low nutrient environments and in the presence of chlorine [40] or monochloramine [41]. However, a challenge with qPCR is that it enumerates both viable and killed intact cells. The discrepancies in results obtained between different *Legionella* detection methods make risk assessment extremely difficult. This is because of a lack of comparability in *Legionella* detection during different studies [39]. Some studies have demonstrated that qPCR coupled with ethidium monoazide (EMA) or propidium monoazide (PMA) pre-treatment enables quantification of only viable cells [42,43,44,45]. EMA and PMA are light activated compounds which bind to free DNA not protected by a cell wall and hence allows only DNA contained within intact cells to be amplified by qPCR. The difficulty with these methods is that the concentration of EMA or PMA has to be optimised for the total DNA present in a sample which is unfeasible for environmental samples of unknown concentration. Studies have shown if the concentration of EMA is too high it may penetrate intact cell, potentially resulting in false negatives [46,47]. Alternatively, insufficient concentration may result in free DNA remaining unbound, causing false positive results [48].

A statistically significant increase in the concentration of *Legionella*, *L. pneumophila* or along the pipeline was observed 2/16 times in DS1 and 4/16 for DS2. This suggests that although there is a potential for amplification of these organisms within the system it is not commonplace. Statistically significantly increases in an organisms’ concentrations occurred sporadically, with low disinfectant residual the only reoccurring accompanying trend. The detected and demonstrable increases in concentrations of an organism at certain points along the distribution network could be due to a number of factors. These might include: the inclusion of a biofilm fragment in the water sample; the accumulation of organisms due to a dead-end; contamination through breaks cracks and joints in the pipeline, or multiplication of organisms [49]. An inclusion of a biofilm fragment could explain the significant increase observed at DS1 point C during summer, as the increase was not observed further down the distribution line at point D. This is supported by the large standard deviation would could be cause by variation in biofilm fragments within water samples. A log_10_ decrease in MAC copies/mL was also observed from point C to D in DS2 during autumn and winter; however, this was not statistically significant due to the large variation in samples and could also be attributed to the inherent variation observed with environmental water samples and the possible addition of biofilm fragments.

The correlation of increases in microbial concentration with low disinfectant residual was expected due to replication. Further research may be required to confirm this opinion. Significant increases due to the inclusion of a biofilm fragment, still represents a result of public health significance. In this instance the multiplication within the biofilm indicates viability and persistence, and the sporadic release of concentrations of public health concern. Sample point E in DS2 was a dead-end, the increased concentrations of *Legionella* observed here demonstrated that dead-ends are clearly of concern. The increase in detected *Legionella* copies may be due to low flow rate, low turbulence resulting in the quenching of disinfectant residual by biofilm and organic matter within the dead-end. Further investigation may determine the relative influence of these factors.

The concentration of *Legionella* spp. measured at sample point A in DS2 during the autumn sampling period has been highlighted in Table 2 as it was a magnitude higher than the concentrations measured at the sampling points further down the pipeline. This increase was assumed likely to be due to the inclusion of a biofilm fragment in the water sample. It highlights the difficulty of measuring the concentration of an organism in environmental water sample which may include biofilm fragments.

The presence of *Legionella* and MAC in South Australian potable water supports the work by Wang *et al.* [2] who used qPCR to detect *Legionella* spp. and *Mycobacterium* spp. from point of use domestic potable water taps in the USA. The highest concentrations of *Legionella*, *L. pneumophila* and *Mycobacterium* spp. detected by Wang *et al.* [2] was 10^3^, 10^1^ and 10^5^ copies/mL respectively, which was comparable with the highest concentrations found in these two distribution pipelines.

## 5. Conclusions

This study confirms the presence of opportunistic pathogens *Legionella* spp., *L. pneumophila* and MAC in both a chlorine and a chloramine disinfected potable water distribution system. The concentrations of these opportunistic pathogens were primarily controlled throughout the distribution network through maintenance of disinfection residuals. However, at a dead-end and when the disinfection residual was not maintained, the pathogens were able to significantly increase in concentration. The potential for dead-ends in pipes to promote growth warrants more attention in efforts to control *Legionella*, *L. pneumophilia* and MAC within these environments. The public health significance of these increases in *Legionella*, *L. pneumophila* and MAC is challenging to assess due to the difficulties with interpreting qPCR results. Improved detection methods will result in a better understanding of the environmental factors influencing colonisation of these systems. The increases in concentrations also demonstrated that these opportunistic pathogens have the potential to survive both disinfection processes. This may have important implications for control along the distribution network and at point of use, particularly in large buildings that may require water to be stored or piped a considerable distance prior to usage.

## References

[1] Lehtola M.J., Torvinen E., Kusnetsov J., Pitkänen T., Maunula L., von Bonsdorff C.-H., Martikainen P.J., Wilks S.A., Keevil C.W., Miettinen I.T. (2007). Survival of *Mycobacterium avium*, *Legionella pneumophila*, *Escherichia coli*, and Caliciviruses in drinking water-associated biofilms grown under high-shear turbulent flow. Appl. Environ. Microbiol..

[2] Wang H., Edwards M.A., Falkinham J.O., Pruden A. (2012). Molecular survey of occurrence of *Legionella* spp., *Mycobacterium* spp., *Pseudomonas aeruginosa* and amoeba hosts in two chloraminated drinking water distribution systems. Appl. Environ. Microbiol..

[3] Szewzyk U., Szewzyk R., Manz W., Schleifer K.-H. (2000). Microbiological safety of drinking water. Ann. Rev. Microbiol..

[4] Stevens M., Ashbolt A., Cunliffe D. (2003). Review of Coliforms as Microbial Indicators of Drinking Water Quality.

[5] Hörman A., Rimhanen-Finne R., Maunula L., von Bonsdorff C.-H., Torvela N., Heikinheimo A., Hänninen M.-L. (2004). *Campylobacter* spp., *Giardia* spp., *Cryptosporidium* spp., noroviruses, and indicator organisms in surface water in southwestern Finland, 2000–2001. Appl. Environ. Microbiol..

[6] Harwood V.J., Levine A.D., Scott T.M., Chivukula V., Lukasik J., Farrah S.R., Rose J.B. (2005). Validity of the indicator organism paradigm for pathogen reduction in reclaimed water and public health protection. Appl. Environ. Microbiol..

[7] Hsu S.C., Martin R., Wentworth B.B. (1984). Isolation of *Legionella* species from drinking water. Appl. Environ. Microbiol..

[8] Buchbinder S., Trebesius K., Heesemann J.R. (2002). Evalution of detection of *Legionella* spp. in water samples by fluorescence *in situ* hybridization, PCR amplification and bacterial culture. Int. J. Med. Microbiol..

[9] Tobin J.O., Dunnill M.S., French M., Morris P.J., Beare J., Fisher-Hoch S., Mitchell R.G., Muers M.F. (1980). Legionnaires’ diseases in a transplant unit: Isolation of the causative agent from shower baths. Lancet.

[10] Neil K., Berkelman R. (2008). Increasing incidence of legionellosis in the United States, 1990–2005: Changing epidemiologic trends. Clin. Infect. Dis..

[11] Centers for Disease Control and Prevention (2013). Surveillance for Waterborne disease outbreaks associated with drinking water and other nonrecreational water—United States, 2009–2010. MMWR.

[12] Schulze-Robbecke R., Janning B., Fischeder R. (1992). Occurrence of mycobacteria in biofilm samples. Tuber. Lung Dis..

[13] Thomson R.M., Carter R., Tolson C., Coulter C., Huygens F., Hargreaves M. (2013). Factors associated with the isolation of Nontuberculous mycobacteria (NTM) from a large municipal water sys in Brisbane, Australia. BMC Microbiol..

[14] Thomson R.M. (2010). Changing epidemiology of pulmonary nontuberculous mycobacteria infections. Emerg. Infect. Dis..

[15] Shin S.J., Lee B.S., Koh W.-J., Manning E.J.B., Anklam K., Sreevatsan S., Lambrecht R.S., Collins M.T. (2010). Efficient differentiation of *Mycobacterium avium* complex species and subspecies by use of five-target multiplex PCR. J. Clin. Microbiol..

[16] O’Brien D.P., Currie B.J., Krause V.L. (2000). Nontuberculous mycobacterial disease in northern Australia: A case series and review of the literature. Clin. Infect. Dis..

[17] Field S.K., Fisher D., Cowie R.L. (2004). *Mycobacterium avium* complex pulmonary disease in patients without HIV infection. Chest.

[18] Huang J.H., Kao P.N., Adi V., Ruoss S.J. (1999). *Mycobacterium avium*-intracellulare pulmonary infection in HIV-negative patients without preexisting lung disease. Chest.

[19] Lakhanpal A., Arfon S., McKeon D.J. (2011). So, they thought it was all over. BMJ Case Rep..

[20] Marras T.K., Wallace R.J., Koth L.L., Stulbarg M.S., Cowl C.T., Daley C.L. (2005). Hypersensitivity pneumonitis reaction to *Mycobacterium* avium in household water. Chest.

[21] Sugita Y. (2000). Familial cluster of cutaneous *Mycobacterium avium* infection resulting from use of a circulating, constantly heated bath water system. Br. J. Dermatol..

[22] Karakousis P.C., Moore R.D., Chaisson R.E. (2004). *Mycobacterium avium* complex in patients with HIV infection in the era of highly active antiretroviral therapy. Lancet Infect. Dis..

[23] Thegerstrom J., Romanus V., Friman V., Brudin L., Haemig P., Olsen B. (2008). *Mycobacterium avium* lymphadenopathy among children, Sweden. Emerg. Infect. Dis..

[24] Nightingale S.D., Byrd L.T., Southern P.M., Jockusch J.D., Cal S.X., Wynne B.A. (1992). Incidence of *Mycobacterium avium*-intracellulare complex bacteremia in human immunodeficiency virus-positive patients. J. Infect. Dis..

[25] Naser S.A., Ghobrial G., Romero C., Valentine J.F. (2004). Culture of *Mycobacterium avium* subspecies paratuberculosis from the blood of patients with Crohn’s disease. Lancet.

[26] Whiley H., Keegan A., Giglio S., Bentham R. (2012). *Mycobacterium avium* complex—The role of potable water in disease transmission. J. Appl. Microbiol..

[27] Von Reyn C.F., Marlow J.N., Arbeit R.D., Barber T.W., Falkinham J.O. (1994). Persistent colonisation of potable water as a source of *Mycobacterium avium* infection in AIDS. Lancet.

[28] Aronson T. (1999). Comparison of large restriction fragments of *Mycobacterium avium* isolates recovered from AIDS and non-AIDS patients with those of isolates from potable water. J. Clin. Microbiol..

[29] Tobin-D’Angelo M.J., Blass M.A., del Rio C., Halvosa J.S., Blumberg H.M., Horsburgh C.R. (2004). Hospital water as a source of *Mycobacterium avium* complex isolates in respiratory specimens. J. Infect. Dis..

[30] Falkinham J.O. (2011). Nontuberculous mycobacteria from household plumbing of patients with nontuberculous mycobacteria disease. Emerg. Infect. Dis..

[31] NHMRC N. (2011). Australian Drinking Water Guidelines.

[32] Giglio S., Monis P.T., Saint C.P. (2005). *Legionella* confirmation using real-time PCR and SYTO9 is an alternative to current methodology. Appl. Environ. Microbiol..

[33] Giglio S., Monis P.T., Saint C.P. (2003). Demonstration of preferential binding of SYBR Green I to specific DNA fragments in real-time multiplex PCR. Nucleic Acids Res..

[34] Park H., Jang H., Kim C., Chung B., Chang C., Park S.K., Song S. (2000). Detection and identification of *Mycobacteria* by amplification of the internal transcribed spacer regions with genus- and species-specific PCR primers. J. Clin. Microbiol..

[35] Livak K.J. (2001). Analysis of relative gene expression data using real-time quantitative PCR and the 2-[Delta][Delta] CT method. Methods.

[36] Staroscik A. Calculator for Determining the Number of Copies of a Template. http://cels.uri.edu/gsc/cndna.html.

[37] Bartram J., Chartier Y., Lee J.V., Pond K., Surman-Lee S. (2007). Legionella and the Prevention of Legionellosis.

[38] Nuzzo R. (2014). Scientific method: Statistical errors *p* values, the “gold standard” of statistical validity, are not as reliable as many scientists assume. Nature.

[39] Whiley H., Taylor M. (2014). Legionella detection by culture and qPCR: Comparing apples and oranges. Crit. Rev. Microbiol..

[40] Chang C.W., Hwang Y.H., Cheng W.Y., Chang C.P. (2007). Effects of chlorination and heat disinfection on long-term starved *Legionella pneumophila* in warm water. J. Appl. Microbiol..

[41] Alleron L., Merlet N., Lacombe C., Frère J. (2008). Long-term survival of *Legionella pneumophila* in the viable but nonculturable state after monochloramine treatment. Curr. Microbiol..

[42] Chang B., Sugiyama K., Taguri T., Amemura-Maekawa J., Kura F., Watanabe H. (2009). Specific detection of viable *Legionella* cells by combined use of photoactivated ethidium monoazide and PCR/real-time PCR. Appl. Environ. Microbiol..

[43] Delgado-Viscogliosi P., Solignac L., Delattre J.-M. (2009). Viability PCR, a culture-independent method for rapid and selective quantification of viable *Legionella pneumophila* Cells in environmental water samples. Appl. Environ. Microbiol..

[44] Yáñez M.A., Nocker A., Soria-Soria E., Múrtula R., Martínez L., Catalán V. (2011). Quantification of viable Legionella pneumophila cells using propidium monoazide combined with quantitative PCR. J. Microbiol. Methods.

[45] Qin T., Tian Z., Ren H., Hu G., Zhou H., Lu J., Luo C., Liu Z., Shao Z. (2012). Application of EMA-qPCR as a complementary tool for the detection and monitoring of Legionella in different water systems. World J. Microbiol. Biotechnol..

[46] Flekna G., Štefanič P., Wagner M., Smulders F.J.M., Možina S.S., Hein I. (2007). Insufficient differentiation of live and dead *Campylobacter jejuni* and * Listeria monocytogenes* cells by ethidium monoazide (EMA) compromises EMA/real-time PCR. Res. Microbiol..

[47] Kobayashi H., Oethinger M., Tuohy M.J., Hall G.S., Bauer T.W. (2009). Unsuitable distinction between viable and dead *Staphylococcus aureus* and *Staphylococcus epidermidis* by ethidium bromide monoazide. Lett. Appl. Microbiol..

[48] Fittipaldi M., Codony F., Adrados B., Camper A., Morató J. (2011). Viable real-time PCR in environmental samples: Can all data be interpreted directly?. Microb. Ecol..

[49] Robertson W., Stanfield G., Howard G., Bartram J. (2006). Monitoring the Quality of Drinking Water during Storage and Distribution. Water Sanitation and Health.

