# Correction: Whiley, H., *et al.* Detection of *Legionella, L. pneumophila* and Mycobacterium Avium Complex (MAC) along Potable Water Distribution Pipelines. *Int. J. Environ.* *Res. Public Health* 2014, *11*, 7393–7405

**DOI:** 10.3390/ijerph111111418

**Published:** 2014-11-03

**Authors:** Harriet Whiley, Alexandra Keegan, Howard Fallowfield, Richard Bentham

**Affiliations:** 1 Health and the Environment, Flinders University, GPO Box 2100, Adelaide 5001, Australia; E-Mails: Howard.Fallowfield@flinders.edu.au (H.F.); Richard.Bentham@flinders.edu.au (R.B.); 2 South Australian Water Corporation, 250 Victoria Square, Adelaide 5000, Australia; E-Mail: Alex.Keegan@sawater.com.au

The authors wish to add the following amendments and corrections to their paper published in *IJERPH* [1].

Page 7398, Table 1: The average water temperature measured in summer is 24.3 °C not 4.3 °C. The correct Table 1 should therefore be:

**Table 1:** Average concentration of *Legionella* spp. *L. pneumophila* and MAC (mean ± standard deviation copies/mL) measured at each sampling point of Distribution system 1 (DS1) using qPCR. Total and free chlorine (mg/L) measured when samples were collected is also shown as well as the average water temperature for the month during which the sample was taken. The sampling points where a significant increase (*p* < 0.05) in the concentration of an organism compared to the concentration measured at sample point A within the same sampling period are also highlighted (*).

Season Sampled and Average Water Temperature	Sample Point	A	B	C	D	E
	Distance from Treatment Plant (km)	5	7	10	18	22
Summer 24.3 °C (*n* = 8)	Total Chlorine (mg/L)	1.4	0.7	1.1	0.4	0.4
	Free chlorine (mg/L)	1.2	0.6	0.1	0.2	0.2
	Average *Legionella* spp. (copies/mL)	37 ± 53	9 ± 4	3 ± 0	187 ± 22	^+^ 1238 ± 47
	Average *L.* *pneumophila* (copies/mL)	10 ± 8	3 ± 0	3 ± 0	375 ± 305	* 1981 ±298
	Average MAC (copies/mL)	36 ± 19	42 ± 19	* 31,813 ± 17,017	116 ± 118	^+^ 4395 ± 2176
Autumn 18.6 °C (*n* = 8)	Total chlorine (mg/L)	1.5	N/A	1.0	0.8	1.1
	Free chlorine (mg/L)	1.3	N/A	0.8	0.6	0.9
	Average *Legionella* spp. (copies/mL)	5 ± 4	N/A	41 ± 21	47 ± 13	46 ± 17
	Average *L.* *pneumophila* (copies/mL)	3 ± 0	N/A	3 ± 0	46 ± 68	^+^ 487 ± 406
	Average MAC (copies/mL)	25 ± 0	N/A	25 ± 0	200 ± 157	25 ± 0
Winter 13.6 °C (*n* = 8)	Total chlorine (mg/L)	1.2	N/A	1.3	0.4	0.7
	Free chlorine (mg/L)	1.1	N/A	1.3	0.3	0.6
	Average *Legionella* spp. (copies/mL)	22 ± 31	N/A	3 ± 0	5 ± 1	3 ± 0
	Average *L.* *pneumophila* (copies/mL)	81 ± 134	N/A	3 ±1	3 ± 0	3 ± 0
	Average MAC (copies/mL)	25 ±0	N/A	25 ± 0	25 ± 0	25 ± 0
Spring 20.3 °C (*n* = 8)	Total chlorine (mg/L)	1.0	0.7	0.3	0.4	0.7
	Free chlorine (mg/L)	0.9	0.6	0.2	0.3	0.6
	Average *Legionella* spp. (copies/mL)	43 ± 69	120 ± 151	8 ± 3	93 ± 139	6 ± 6
	Average *L.* *pneumophila* (copies/mL)	36 ± 34	15 ± 12	9 ± 1	166 ± 122	3 ± 0
	Average MAC (copies/mL)	2468 ± 317	2224 ± 2342	1112 ± 328	294 ± 58	101 ± 69

N/A sample was not available to be collected at this time. * statistically significant increase. ^+^ an increase of concentration by an order of magnitude. The lack of statistical significance (*p* > 0.05) is possible due to the large variance in environmental samples shown by the standard deviation.

The authors would like to apologize for any inconvenience caused to the readers by these changes.

## References

[1] Whiley H., Keegan A., Fallowfield H., Bentham R. (2014). Detection of *Legionella*, *L. pneumophila* and Mycobacterium Avium Complex (MAC) along potable water distribution pipelines. Int. J. Environ. Res. Public Health.

